# CRISPR-Cas System, Antimicrobial Resistance, and *Enterococcus* Genus—A Complicated Relationship

**DOI:** 10.3390/biomedicines12071625

**Published:** 2024-07-22

**Authors:** Carmen Costache, Ioana Colosi, Dan-Alexandru Toc, Karla Daian, David Damacus, Alexandru Botan, Adelina Toc, Adrian Gabriel Pana, Paul Panaitescu, Vlad Neculicioiu, Pavel Schiopu, Dumitrana Iordache, Anca Butiuc-Keul

**Affiliations:** 1 Department of Microbiology, Iuliu Hatieganu University of Medicine and Pharmacy, 400012 Cluj-Napoca, Romaniaicolosi@umfcluj.ro (I.C.);; 2 Cluj County Emergency Hospital, 400000 Cluj-Napoca, Romania; 3 Faculty of Medicine, Iuliu Hatieganu University of Medicine and Pharmacy, 400012 Cluj-Napoca, Romania; 4 Department of Molecular Biology and Biotechnology, Faculty of Biology and Geology, Babeş-Bolyai University, 400084 Cluj-Napoca, Romania; 5 Centre for Systems Biology, Biodiversity and Bioresources, Babes-Bolyai University, 400006 Cluj-Napoca, Romania

**Keywords:** antibiotic resistance, CRISPR, CRISPRCasFinder, *efmA*, *Enterococcus* genus, *IsaE*, *vanA*, *vanM*, *Enterococcus faecalis*, *Enterococcus faecium*

## Abstract

(1) Background: The rise in antibiotic resistant bacteria poses a significant threat to public health worldwide, necessitating innovative solutions. This study explores the role of Clustered Regularly Interspaced Short Palindromic Repeats (CRISPR) in the context of antibiotic resistance among different species from the *Enterococcus* genus. (2) Methods: The genomes of *Enterococcus* included in the study were analyzed using CRISPRCasFinder to distinguish between CRISPR-positive (level 4 CRISPR) and CRISPR-negative genomes. Antibiotic resistance genes were identified, and a comparative analysis explored potential associations between CRISPR presence and antibiotic resistance profiles in *Enterococcus* species. (3) Results: Out of ten antibiotic resistance genes found in *Enterococcus* species, only one, the *efmA* gene, showed a strong association with CRISPR-negative isolates, while the others did not significantly differ between CRISPR-positive and CRISPR-negative *Enterococcus* genomes. (4) Conclusion: These findings indicate that the *efmA* gene may be more prevalent in CRISPR-negative *Enterococcus* genomes, and they may contribute to a better understanding of the molecular mechanisms underlying the acquisition of antibiotic resistance genes in *Enterococcus* species.

## 1. Introduction

The *Enterococcus* genus is a group of Gram-positive, facultatively anaerobic bacteria, commonly present in surface waters, soil, and even in the human and animal gastrointestinal tracts [1]. Isolated from water, they can be an indicator of fecal contamination, which is the result of the bacteria’s ubiquity in various environments [2]. Some of the *Enterococcus* species are harmless or even play essential roles relate to the host’s health, while others are opportunistic pathogens, hospitalized patients being the ones that contract infections more often [3].

Within the *Enterococcus* genus, *E. faecalis* and *E. faecium* are the most clinically relevant species, being commonly found in the gastrointestinal tract [4]. There are *Enterococcus* species that are not common and are known as “other enterococci (OE)”, this group being represented by *E. avium*, *E. casseliflavus*, *E. durans*, *E. gallinarum*, *E. mundtii*, and *E. raffinosus* [5]. The OE group is divided into two subgroups: the *vanC* subgroup, which is characterized by chromosomally encoded vancomycin-resistance genes and includes *E. casseliflavus* and *E. gallinarum*, and the non-*vanC* subgroup, which includes the other species of enterococci and consists of acquired resistance genes to vancomycin through mobile genetic elements [6].

CRISPR-Cas is a defense system that has been developed by bacteria to protect themselves against viruses and other foreign genetic elements. This system consists of Clustered Regularly Interspaced Short Palindromic Repeats (CRISPR) and associated genes for an endonuclease called CRISPR-associated protein (*cas)*, which can integrate fragments of foreign nucleic acids from viruses and mobile genetic elements into the CRISPR array [7]. For the first time, in 1980, repetitive sequences were observed in the *E. coli* genome [8].

The CRISPR-Cas system is classified into 2 classes, 6 types, and 33 subtypes. Class 1 includes types I, II, and IV, while class 2 includes types III, V, and VI [9].

There are cases where bacteria may have *cas* genes but lack the CRISPR arrays. Even if a bacterium lacks CRISPR arrays, it can still be classified within a particular CRISPR-Cas type based on the organization of the Cas proteins [9].

There are several databases and tools available for CRISPR research and analysis, some of them being CRISPRCasdb (https://crisprcas.i2bc.paris-saclay.fr/, accessed on 17 July 2024), CRISPR-Cas9 Target Finder (https://flycrispr.org/target-finder/, accessed on 17 July 2024), CRISPRminer (http://www.microbiome-bigdata.com/CRISPRminer/, accessed on 17 July 2024), or CRISPRone (https://omics.informatics.indiana.edu/CRISPRone/, accessed on 17 July 2024). These databases provide valuable resources for studying and exploring the diversity and functions of CRISPR-Cas systems.

The CRISPR-Cas9 system has many applications as a gene editor as well as in the development of defense systems against resistant bacteria. Therefore, the system can be used in the treatment of various conditions, including cancer, genetic disorders (e.g., sickle cell, beta thalassemia, and others), infections (e.g., HIV) [10], Parkinson’s disease, diabetes mellitus, or Alzheimer’s disease [11]. 

By engineering bacteriophages with CRISPR-Cas, it is possible to create phages that can precisely target and disrupt specific genes in the bacterial genome. This approach can be particularly useful in targeting antibiotic resistance genes in pathogenic bacteria, thereby making them susceptible to treatment again [12]. Phage delivery of CRISPR-Cas antimicrobials is thought to be the most promising method currently available [13].

As a result of the extensive use of antibiotics, a large number of bacterial strains have developed defense mechanisms that lead to their resistance to antibiotics. Antibiotic resistance is primarily achieved through the transfer of specific resistance genes (ARGs) with the help of mobile genetic elements (MGEs), such as plasmids and integrons. The acquisition of ARGs by bacteria is accomplished through Horizontal Gene Transfer (HGT). The CRISPR-Cas system interferes with HGT and can prevent the transfer of ARGs [14]. As a result, bacteria with nonfunctional CRISPR-Cas systems are less likely to acquire foreign DNA, such as ARGs [15]. A nonfunctional CRISPR-Cas system refers to a system that is unable to perform its normal biological functions because it has lost his functions due to various reasons [16]. 

Previous studies showed that MDR *Enterococcus* strains usually lack an active CRISPR-Cas system [17]. Price et al. showed that the lack of a CRISPR-Cas system has a significant impact on the way conjugative plasmids behave in a biofilm setting [18]. Enterococci can harbor a variety of antibiotic resistance genes, allowing them to resist the action of many commonly used antibiotics [19]. Depending on the antibiotic resistance gene pattern, they can be resistant to vancomycin, aminoglycosides, tetracyclines, macrolides, chloramphenicol, clindamycin, or beta-lactams [20].

The acquired glycopeptide resistance in *Enterococcus* is linked to the presence of various genes, such as *vanA*, *vanB*, *vanD*, *vanE*, *vanG*, *vanL*, *vanM*, or *vanN* [21]. Among these, the *vanA* gene is the most prevalent and encodes enzymes involved in altering the bacterial cell wall structure, being responsible for resistance to not only vancomycin, but also to teicoplanin [22]. In contrast, the *vanB* gene is responsible for only vancomycin resistance, these enterococci remaining susceptible to teicoplanin. The *vanM* gene, along with *vanR*, *vanS*, *vanH*, *vanY*, and *vanX* genes, are referred to as the *vanM* cluster [23].

Another antibiotic-resistance-related gene is the *efmA* gene, which encodes an efflux pump directed to macrolides, expelling erythromycin and related antibiotics from the bacterial cell [24]. The same mechanism is available for the *msrC* gene, which encodes a macrolide-specific efflux pump [25].

The *IsaE* gene is responsible for resistance to lincosamides [26] and the *InuG* gene confers resistance to both lincosamide and streptogramin B [27].

Antibiotic resistance in *Enterococcus* species is a major global health challenge, making the treatment of enterococcal infections increasingly difficult. In this context, limiting the antibiotic resistance benefitting by the CRISPR-Cas system in *Enterococcus* species has been an area of growing interest [28].

This study aims to shed light on the role of CRISPR-Cas systems in *Enterococcus* antibiotic resistance and contribute to understanding the complex interplay between CRISPR-Cas systems and HGT of ARGs.

## 2. Materials and Methods

### 2.1. Identification of Enterococcus Isolates

The genomes of the *Enterococcus* isolates were obtained from the CRISPRCasdb, which is an online database that provides valuable information about CRISPR-Cas systems identified in various prokaryotic organisms. It consists of two programs: CRISPRCasFinder, used to detect CRISPRs and *cas* genes, and database tools [29]. The database was filtered to include only *Enterococcus* species with level 4 CRISPR arrays, which were considered CRISPR-positive. Isolates with lower CRISPR levels were considered CRISPR-negative, creating two groups: CRISPR-positive and CRISPR-negative.

The CRISPR arrays were classified in levels from 1 to 4, based on the structure of CRISPR array found in each isolate. The lowest levels represent the CRISPRs with fewer than four spacers and three or more perfect repeats. In a real CRISPR array, the conservation of repeats must be high and the similarity between spacers must be low. Level 4 CRISPR arrays are considered, in our study, CRISPR-positive, because they are the most reliable ones [29].

### 2.2. Genome Retrieval and Database Generation

For each *Enterococcus* isolate, the corresponding chromosome and, if applicable, plasmid sequences were retrieved from the database and were downloaded and compiled into a database using the BioEdit software, version 7.2.6. This database of genomes served as the basis for further analyses. The information obtained from the online database, including species identification, CRISPR status, and Cas type, was organized into a Microsoft Excel spreadsheet along with annotations for each *Enterococcus* isolate, resulting in a total of 280 *Enterococcus* strains. To validate the accuracy of the genome sequences and assess the genetic similarity among isolates, a Basic Local Alignment Search Tool (BLAST) analysis was performed. The downloaded genomes were subjected to BLAST searches within the National Center for Biotechnology Information (NCBI) nucleotide database. The BLAST results were used to confirm the species identity of the isolates and verify the presence of CRISPR arrays and *cas* genes.

**Figure 1:**
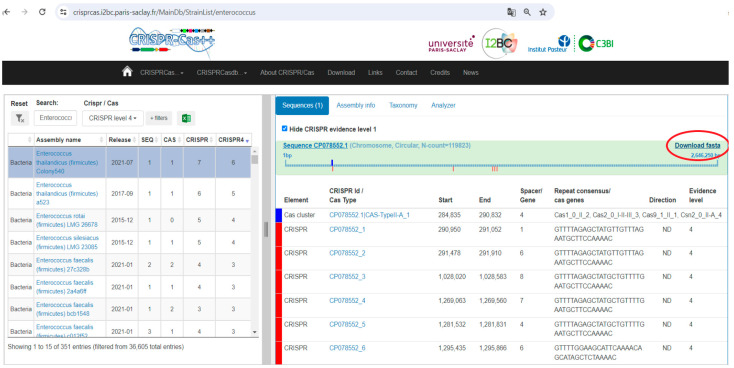
Visual presentation of processing *Enterococcus* genomic data using CRISPRCasdb. The CRISPRCasdb interface shows *Enterococcus* genomes with identified CRISPR-Cas elements, allowing data download.

Figure 1 provides a step-by-step visual guide for processing genomic data from *Enterococcus* isolates using CRISPRCasdb and subsequent software. The steps are:

1. CRISPRCasdb Interface: The genomes of *Enterococcus* isolates are shown with CRISPR-Cas elements identified. The “Download Data” button allows users to download the genomic data.
2. FASTA Format Sequence: Following data retrieval, the acquired information is depicted in the FASTA format, sourced from NCBI’s comprehensive nucleotide database. This format ensures accessibility and compatibility for subsequent analytical steps.
3. Sequence Compilation in BioEdit: In BioEdit, genomes can be introduced by importing sequence data files in various formats. Users typically navigate to the “File” menu and select options such as “Open” or “Import” to load sequence files. Common file formats supported by BioEdit include FASTA, GenBank, and other commonly used sequence file formats. Once imported, the sequences are displayed in the BioEdit workspace, where users can perform various sequence manipulation and analysis tasks. The procured sequence data undergo compilation and comprehensive analysis within the BioEdit software environment. Here, the software showcases the aligned sequences, furnishing researchers with a structured platform for in-depth exploration and investigation.

### 2.3. Identification of Antibiotic Resistance Genes and Statistical Analysis

Through the use of specialized bioinformatics tools and databases, such as CARD (Comprehensive Antibiotic Resistance Database) (https://card.mcmaster.ca/home, accessed on 17 July 2024), version 3.2.4, the antibiotic resistance genes found in the *Enterococcus* isolates were identified through chromosome and, if applicable, plasmid sequence analysis [30]. The number of isolates with antibiotic resistance genes was recorded in a Microsoft Excel for both the CRISPR-positive and CRISPR-negative groups. For each gene, statistical analysis, such as Fisher’s exact test, was performed to evaluate the significance of associations between the presence of the CRISPR-Cas system and antibiotic resistance genes. The level of significance for the Fisher’s exact test was determined to be *p* < 0.05, indicating a statistically significant result.

## 3. Results and Discussion

### 3.1. Comparative Analysis of CRISPR-Positive and CRISPR-Negative Enterococcus Genomes

**Figure 2:**
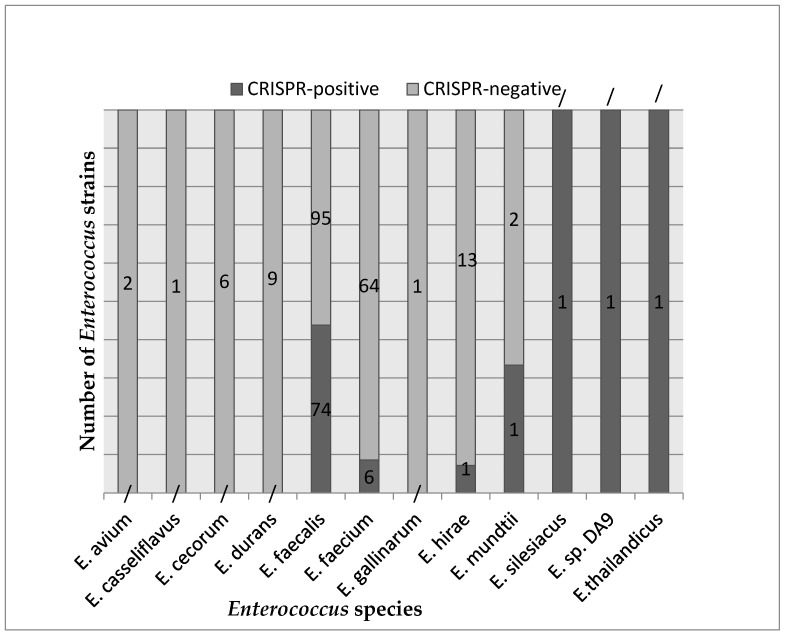
The distribution of the CRISPR-positive and CRISPR-negative isolates among the *Enterococcus* species strains. Each group, CRISPR-positive and CRISPR-negative, contains different *Enterococcus* species (horizontal axis), and for each species, a different number of strains (noted on the columns).

Out of 280 *Enterococcus* isolates, 85 were CRISPR-positive and 195 were CRISPR-negative. Among the 85 CRISPR-positive strains, *E. faecalis* was the most prevalent species (74 strains), followed by *E. faecium* (6 strains), and 1 strain of each of the following: *E. thailandicus*, *E. silesiacus*, *E. hirae*, *E. mundtii*, and *E. sp. DA9*. On the other hand, among the 195 CRISPR-negative strains, *E. faecalis* (95 strains) and E. *faecium* (64 strains) were the most abundant species, along with 9 strains of *E. durans*, 13 strains of *E. hirae*, 2 strains of *E. mundtii*, 6 strains of *E. cecorum*, 2 strains of *E. avium*, 1 strain of *E. casseliflavus*, and 1 strain of *E. gallinarum*. The distribution of Enterecoccus strains is summarized in Figure 2.

The observed distribution of CRISPR-positive and CRISPR-negative strains among *Enterococcus* species highlights potential species-specific variations in CRISPR-Cas systems.

Among the 85 CRISPR-positive strains, three distinct CRISPR-Cas types were identified. The most prevalent type was IIA, present in 65 CRISPR-positive strains, followed by IIC found in 18 strains. Notably, one strain only was characterized by CRISPR-Cas type IC.

**Figure 3:**
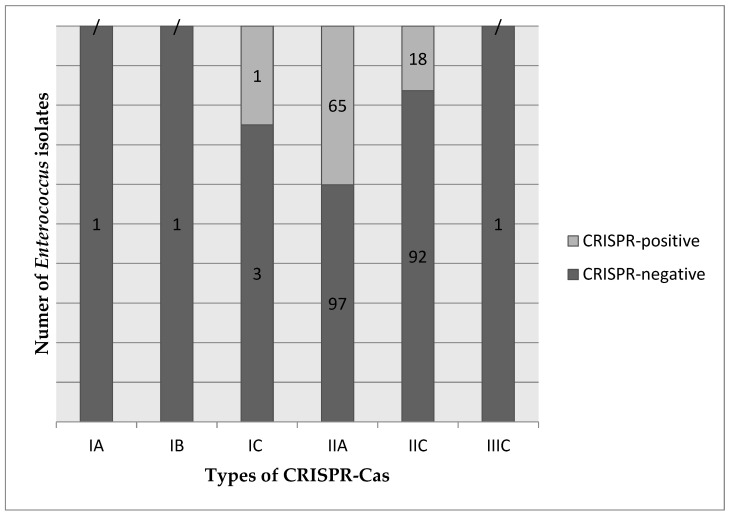
The distribution of the CRISPR-Cas types among the CRISPR-positive and CRISPR-negative isolates. Each group, CRISPR-positive and CRISPR-negative, contains a different number of *Enterococcus* isolates (noted on the columns) for each type (horizontal axis).

Based on the *cas* genes, the CRISPR-negative strains can also be classified into the above-mentioned types. Therefore, among the 195 CRISPR-negative strains, six CRISPR-Cas types were detected. Type IIA was the most common, observed in 97 CRISPR-negative strains, while type IIC was present in 92 strains. There is one isolate for each of the IA, IB, and IIIC types and three strains for the IC type (Figure 3).

Regarding the CRISPR-Cas types, our findings suggest a diverse distribution of CRISPR-Cas types in *Enterococcus* species, with high prevalence of types IIA and IIC. The presence of multiple CRISPR-Cas types enables them to adapt to diverse environmental challenges. These findings align with previous studies that have shown variations in CRISPR-Cas types across different bacterial species and strains [31]. Lyons et al. [31] showed that type II CRISPR1-Cas1 incidence varies significantly between species, CRISPR-Cas distribution being affected by the selective pressure of the environment.

### 3.2. Comparative Analysis of the Antibiotic Resistance Genes Found in the CRISPR-Positive and CRISPR-Negative Isolates

In this study, we assessed a total of 716 sequences of *Enterococcus* (having CRISPR array in chromosome and/or plasmids), of which 187 were classified as CRISPR-positive, while 529 were categorized as CRISPR-negative based on the presence or absence of level 4 CRISPR-Cas systems. 

The large sample size allowed for a comprehensive analysis of the distribution of CRISPR-Cas systems in *Enterococcus* species. These sequences were derived from 280 *Enterococcus* strains, *E. faecalis* being the most prevalent, accounting for 169 strains, followed by *E. faecium* with 70 strains. These findings are consistent with previous reports highlighting the prominence of these two species in clinical settings and their association with various infections [32].

Among the CRISPR-positive isolates, there were identified 7 antibiotic resistance genes, and for the CRISPR-negative isolates there were 10 identified genes, as shown in Table 1.

Among the CRISPR-positive strains, the *vanA* gene was detected in 5 out of 187 genomes (2.7%), while in the CRISPR-negative strains, 9 out of 529 genomes (1.7%) carried the *vanA* gene. The difference in *vanA* gene prevalence between the two groups was not statistically significant (*p* = 0.3774). For the *IsaE* gene, 12 out of 187 CRISPR-positive genomes (6.4%) and 17 out of 529 CRISPR-negative genomes (3.2%) were positive. However, there was no significant association between the *IsaE* gene and CRISPR status (*p* = 0.0812). The *vanM* gene was found in 3 out of 529 CRISPR-negative genomes (0.6%), but none were detected in the CRISPR-positive group (*p* = 0.5713). Similarly, the *InuG* gene was detected in 1 out of 187 CRISPR-positive genomes (0.5%) and 3 out of 529 CRISPR-negative genomes (0.6%), with no significant association (*p* = 1.0000). Regarding the *fexB* gene, it was present in 1 out of 187 CRISPR-positive genomes (0.5%) and 2 out of 529 CRISPR-negative genomes (0.4%), showing no statistically significant association with CRISPR status (*p* = 1.0000). The *fosB3* gene was not found in any of the CRISPR-positive genomes but was present in 2 out of 529 CRISPR-negative genomes (0.4%), and no significant association was observed (*p* = 1.0000). The *AAC(6′)-Iih*, *dfrE*, and *msrC* genes did not show statistically significant association with CRISPR status either. Remarkably, the *efmA* gene exhibited a statistically significant association with CRISPR status. It was detected in only 1 out of 187 CRISPR-positive genomes (0.5%), but was present in 52 out of 529 CRISPR-negative genomes (9.8%) (*p* = 0.00001). These results are highlighted in Table 1 and Table 2.

**Figure 4:**
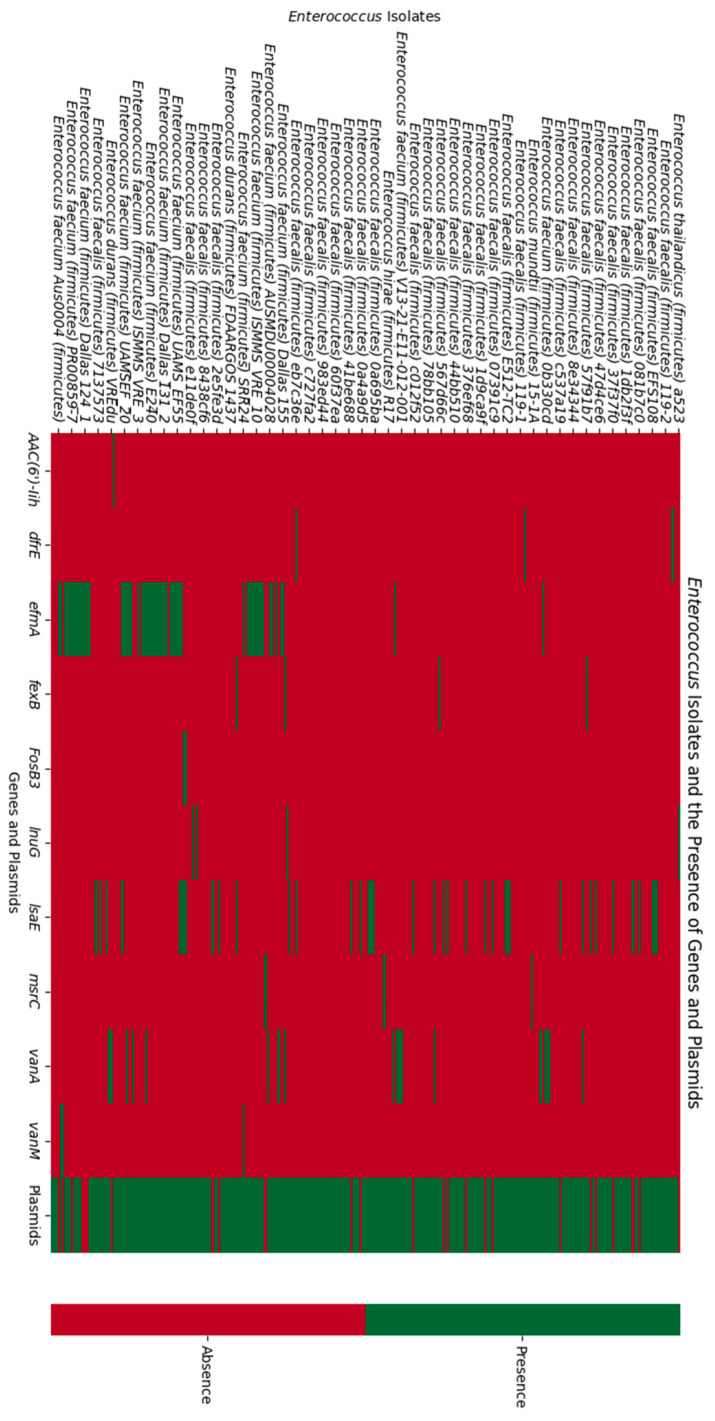
Heatmap showing the relationship between the *Enterococcus* isolates and the presence of genes and plasmids. The vertical axis represents the *Enterococcus* isolates, while the horizontal axis denotes the genes and plasmids. Each cell within the heatmap is color-coded to indicate the presence or absence of the respective genes and plasmids in each isolate. A green color signifies the presence of a gene/plasmid, whereas a red color indicates its absence. The varying patterns of green and red across the heatmap highlight the differences in gene presence among the isolates.

In this study, two heatmaps were generated to visualize the genomic characteristics of various *Enterococcus* isolates. The first heatmap (Figure 4) focuses on the presence of antibiotic resistance genes (ARGs) and plasmids. On the vertical axis, different *Enterococcus* isolates are listed, while the horizontal axis includes ARGs and plasmid presence. The presence of a specific ARG in an isolate is indicated by a green cell, while a red cell denotes its absence. Similarly, plasmid presence is color-coded: green indicates the presence of plasmids, and red indicates their absence. This heatmap allows for a quick and intuitive assessment of the distribution and prevalence of ARGs and plasmids among the *Enterococcus* isolates.

**Figure 5:**
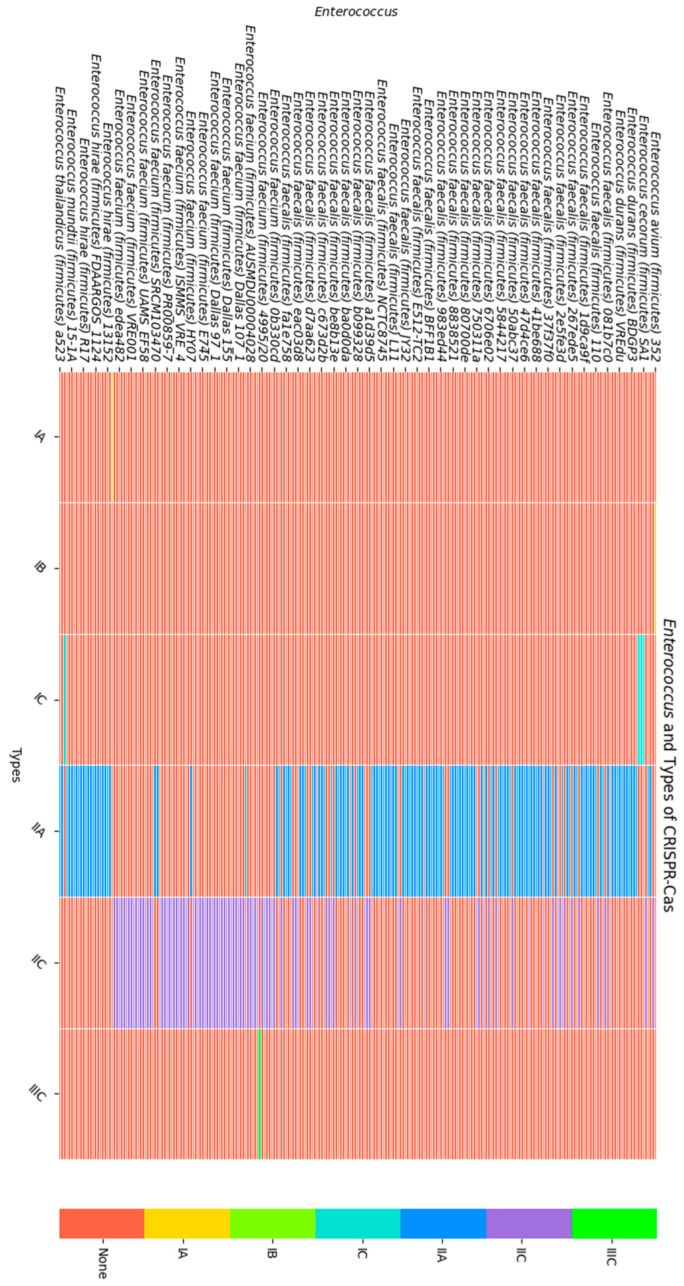
Heatmap showing the relationship between the *Enterococcus* isolates and types of CRISPR-Cas. The vertical axis represents the *Enterococcus* isolates, while the horizontal axis denotes the CRISPR-Cas types. Each cell within the heatmap is color-coded to indicate the presence or absence of the respective types in each isolate (a different color for each type). A red color indicates the absence of the respective type.

The second heatmap (Figure 5) provides information on the types of CRISPR-Cas systems found in the same *Enterococcus* isolates. Again, the vertical axis lists the *Enterococcus* isolates, while the horizontal axis shows the different types of CRISPR-Cas systems. The presence of a specific CRISPR-Cas type is marked by a green cell, and its absence is marked by a red cell. This heatmap enables the visualization of the diversity and distribution of CRISPR-Cas systems across the isolates.

These heatmaps together provide a comprehensive overview of the genomic landscape, highlighting the patterns and trends in antibiotic resistance genes, plasmid presence, and CRISPR-Cas systems.

The analysis revealed that the prevalence of the *vanA*, *vanM*, *IsaE*, *fexB*, *InuG*, *fosB3*, *AAC(6′)-lih*, *dfrE*, and *msrC* genes did not significantly differ between CRISPR-positive and CRISPR-negative *Enterococcus* strains. However, the *efmA* gene showed a strong association with CRISPR-negative strains, indicating that the *efmA* gene may be more prevalent in CRISPR-negative *Enterococcus* strains. Similar data were obtained from Tao S et al. in a study where they analyzed 110 strains of *Enterococcus* [33]. Their study showed an association between the CRISPR-negative strains and two antimicrobial resistance genes, *AAC(6′)-Ii* and *efmA* [33]. However, they mentioned that this observation might have been due to either small sample size or the selected strains not being representative. With an elevated sample size, our study was able to support their initial findings and conclude that there is indeed an association possible between the CRISPR-negative strains and the *efmA* gene.

Among the *E. faecium* isolates, the *efmA* gene presented a statistically significant association with CRISPR status, with 1 out of 24 CRISPR-positive genomes (4.1%) and 52 out of 217 CRISPR-negative genomes (23.9%) (*p* = 0.0341). Moreover, the *vanA* gene presented a statistically significant association with CRISPR status, with 5 out of 24 CRISPR-positive genomes (20.8%) and 9 out of 217 CRISPR-negative genomes (4.1%) (*p* = 0.007), as shown in Table 3. Interestingly, the CRISPR-positive strains presented a significant lower prevalence of antibiotic resistance genes compared to the CRISPR-negative strains, although the significance was observed only in one gene. Tao et al. [28] showed that the distribution of *tetM*, *ermB*, *aadE*, *ant (6)*, and *aac (6′)-aph (2″)* between the CRISPR-negative and the CRISPR-positive isolates was statistically significant (*p* < 0.05) among *E. faecalis* and *E. faecium* strains. Palmer et al. [17] presented a significant distribution for the *tetM* and *ermB* genes (*p* = 0.0003) among *E. faecalis* and Gholizadeh et al. [34] presented a significantly lower distribution of the *tetM*, *ermA*, *ermB*, *vanA*, *aac6′-aph(2″)*, *aadE*, and *ant(6)* genes in CRISPR-positive isolates (*p* < 0.05) among *E. faecalis*. However, in our study, the distribution of ARGs between CRISPR-positive and CRISPR-negative isolates was significant only for the *efmA* gene. These studies suggest that the CRISPR-Cas system might act as a natural barrier against the transmission of antibiotic resistance genes. The results of Pursey et al. [35] and Price VJ et al. [36] also align with these findings. Dos Santos et al. [37] reported an association between the presence of the *vanA* gene and CRISPR among *E. faecalis* strains. However, in our study, this association was only present among *E. faecium* strains.

In our investigation of CRISPR-positive *Enterococcus* isolates, we observed distinct patterns of plasmid presence and the distribution of specific antibiotic resistance genes. We categorized the strains based on their plasmid content, revealing intriguing associations with the prevalence of certain resistance genes.

Among the 85 CRISPR-positive strains, we identified 24 strains that lacked plasmids. Within this group, we found the presence of five *IsaE* genes and one *InuG* gene. Among the 61 strains that contained one or more plasmids, we noted the presence of one *dfrE*, five *IsaE*, one *fexB*, five *vanA*, one *efmA* and one *msrC* gene.

Regarding the 195 CRISPR-negative strains, we identified 55 strains that lacked plasmids. Within this group, there were four *IsaE* genes, five *efmA* genes, one *vanA* gene and one *msrC* gene. Among the 140 strains that contain one or more plasmids, there were 1 *AAC(6′)-Iih* gene, 1 *dfrE* gene, 2 *fexb* genes, 2 *fosB3* genes, 3 *vanM* genes, 3 *InuG* genes, 8 *vanA* genes, 13 *IsaE* genes, and 47 *efmA* genes. A correlation between the presence of plasmids and the presence of antibiotic resistance genes was only shown for the *efmA* gene, which was found in 48 out of 201 isolates containing plasmids (23.8%) and in 5 out of 79 isolates without plasmids (6.3%) (*p* = 0.0006).

The majority of *efmA* genes were discovered in CRISPR-negative plasmids. These findings highlight the significance of HGT as a key mechanism for the dissemination of antibiotic resistance. The ability of plasmids to carry multiple resistance genes can have a substantial effect on the genetic diversity and adaptability of bacterial communities, making them important players in HGT. The rapid spread of antibiotic resistance may be facilitated by the absence of CRISPR-Cas systems in these plasmids, which could allow for the unrestricted transfer and acquisition of resistance genes, aligning with the results of other studies, such as Pinilla-Redondo et al. [38] suggesting the important contribution of plasmids to HGT and high prevalence of CRISPR-Cas systems on plasmids. Pinilla-Redondo et al. [39] also discovered that prokaryotic MGEs—the majority of which are thought to be plasmids—are primarily responsible for encoding type IV CRISPR-Cas system loci. Their findings suggest that, in order to dominate the host environment, plasmid-like elements use type IV systems to eradicate other plasmids with comparable characteristics. According to research by Murugesan et al. [40], many methicillin-resistant *Staphylococcus coagulans* isolates had type IIIA CRISPR-Cas systems and were present within the SCCmec (staphylococcal chromosomal cassette mec) mobile genetic element, demonstrating the involvement of CRISPR-Cas systems in blocking phage/plasmid invasion and horizontal gene transfer of antimicrobial resistance genes. Garneau et al. [41] suggest that *Streptococcus thermophilus* experiences plasmid loss as a result of the CRISPR/Cas system, which offers an easy way to create a strain of bacteria that is resistant to plasmids containing genes for antibiotic resistance.

As previously mentioned, the CRISPR-Cas system may act as an immune effector in fighting the acquisition of foreign DNA from different mobile genetic elements. Price VJ et al. analyzed the behavior of the CRISPR-Cas system in vitro and in vivo and their works showed that perhaps this system works better in vivo rather in vitro [36]. This work highlights an important challenge in using CRISPR-Cas-based approaches to tackle antimicrobial resistance phenomenon in different bacteria. Since these techniques are increasingly studied, we might be facing a lot of different new challenges and thus there is a need to approach them with cautiousness and in a systematic manner [42,43,44]. 

Future applications of CRISPR-Cas technology show great potential in medical and biological sciences. One significant area is the development of CRISPR-based screening tests for antibiotic-resistant strains, such as *Enterococcus*. These tests could quickly identify resistance genes, enabling timely and appropriate treatment decisions. Additionally, CRISPR-Cas could be utilized to design rapid diagnostic tests to detect a wide range of pathogens or genetic conditions. The use of CRISPR-Cas as a target for new medications is another promising application. By focusing on specific genes responsible for disease or antibiotic resistance, new therapeutic strategies can be devised to inhibit these genes, offering more effective treatments. By studying and manipulating the human microbiome using the CRISPR-Cas system, treatments for diseases associated with microbial imbalances, such as inflammatory bowel disease and obesity, could be developed. The potential applications of CRISPR-Cas are continuously expanding. Engaging in interdisciplinary research and collaboration is important for discovering new opportunities. For instance, combining CRISPR-Cas with artificial intelligence or bioinformatics could enhance precision in gene editing.

## 4. Conclusions

Our study provides evidence for a potential association between the CRISPR-Cas system and antibiotic resistance in *Enterococcus* species. CRISPR-positive isolates demonstrated a lower prevalence of antibiotic resistance genes, suggesting that the CRISPR-Cas system may act as a natural barrier against the spread of antibiotic resistance in these bacteria. Although this significant difference is only noticed for the *vanA* gene among *E. faecium* and the *efmA* gene. This phenomenon can be attributed to the likelihood that the CRISPR-Cas system is more active in antiviral protection, with bacteriophages serving as an active regulatory factor in bacterial communities.

These insights contribute to a better understanding of the molecular mechanisms underlying antibiotic resistance in *Enterococcus* and may contribute to the development of targeted strategies to combat multidrug-resistant infections, including in the development of specific bacteriophage therapy, which can be especially helpful in focusing on pathogenic bacteria’s antibiotic resistance genes, making them more susceptible to treatment.

## Figures and Tables

**Table 1:** The distribution of ARGs among CRISPR-negative and CRISPR-positive isolates. Each gene is found in a different number among the two groups, CRISPR-positive and CRISPR-negative.

Gene	CRISPR-Positive (*n* = 187 Sequences)	CRISPR-Negative (*n* = 529 Sequences)
*AAC(6′)-Iih*	0 (0%)	1 (0.19%)
*dfrE*	1 (0.53%)	1 (0.19%)
*efmA*	1 (0.53%)	52 (9.83%)
*fexB*	1 (0.53%)	2 (0.38%)
*FosB3*	0 (0%)	2 (0.38%)
*lnuG*	1 (0.53%)	3 (0.57%)
*lsaE*	12 (6.42%)	17 (3.21%)
*msrC*	1 (0.53%)	1 (0.19%)
*vanA*	5 (2.67%)	9 (1.70%)
*vanM*	0 (0%)	3 (0.57%)

**Table 2:** The table presents the results of Fisher’s exact test for the ARGs. Only for the *efmA* gene is the test statistically significant (*p* < 0.05), which is represented in green.

Gene	*p* (Fisher’s Exact Test)
*efmA*	*p* < 0.05
*fexB*	1
*FosB3*	1
*lnuG*	1
*lsaE*	0.0812
*vanA*	0.3774
*vanM*	0.5713

**Table 3:** Distribution (%) of the *efmA* and *vanA* genes among CRISPR-positive and CRISPR-negative isolates in *E. faecium*.

*E. faecium*	CRISPR-Positive	CRISPR-Negative	*p* (Fisher’s Exact Test)
*efmA*	4.1%	23.9%	0.0034
*vanA*	20.8%	4.1%	0.007

## Data Availability

Data is contained within the article.

## References

[1] García-Solache M., Rice L.B. (2019). The Enterococcus: A Model of Adaptability to Its Environment. Clin. Microbiol. Rev..

[2] Torres C., Alonso C.A., Ruiz-Ripa L., León-Sampedro R., Del Campo R., Coque T.M. (2018). Antimicrobial Resistance in *Enterococcus* spp. of animal origin. Microbiol. Spectr..

[3] Fiore E., Van Tyne D., Gilmore M.S. (2019). Pathogenicity of Enterococci. Microbiol. Spectr..

[4] Kim M.-A., Rosa V., Min K.-S. (2020). Characterization of *Enterococcus faecalis* in different culture conditions. Sci. Rep..

[5] Toc D.A., Pandrea S.L., Botan A., Mihaila R.M., Costache C.A., Colosi I.A., Junie L.M. (2022). *Enterococcus raffinosus*, *Enterococcus durans* and *Enterococcus avium* Isolated from a Tertiary Care Hospital in Romania—Retrospective Study and Brief Review. Biology.

[6] Monticelli J., Knezevich A., Luzzati R., Di Bella S. (2018). Clinical management of non-*faecium* non-*faecalis* vancomycin-resistant enterococci infection. Focus on *Enterococcus gallinarum* and *Enterococcus casseliflavus*/*flavescens*. J. Infect. Chemother..

[7] Butiuc-Keul A., Farkas A., Carpa R., Iordache D. (2022). CRISPR-Cas System: The Powerful Modulator of Accessory Genomes in Prokaryotes. Microb. Physiol..

[8] Ishino Y., Krupovic M., Forterre P. (2018). History of CRISPR-Cas from Encounter with a Mysterious Repeated Sequence to Genome Editing Technology. J. Bacteriol..

[9] Iordache D., Baci G.-M., Căpriță O., Farkas A., Lup A., Butiuc-Keul A. (2022). Correlation between CRISPR Loci Diversity in Three Enterobacterial Taxa. Int. J. Mol. Sci..

[10] Wang H., La Russa M., Qi L.S. (2016). CRISPR/Cas9 in Genome Editing and Beyond. Annu. Rev. Biochem..

[11] Barrangou R., Sontheimer E.J., Marraffini L.A. (2022). Crispr: Biology and Applications.

[12] Khambhati K., Bhattacharjee G., Gohil N., Dhanoa G.K., Sagona A.P., Mani I., Bui N.L., Chu D., Karapurkar J.K., Jang S.H. (2023). Phage engineering and phage-assisted CRISPR-Cas delivery to combat multidrug-resistant pathogens. Bioeng. Transl. Med..

[13] Duan C., Cao H., Zhang L.-H., Xu Z. (2021). Harnessing the CRISPR-Cas Systems to Combat Antimicrobial Resistance. Front. Microbiol..

[14] Tao S., Chen H., Li N., Liang W. (2022). The Application of the CRISPR-Cas System in Antibiotic Resistance. Infect. Drug Resist..

[15] Marraffini L.A., Sontheimer E.J. (2008). CRISPR Interference Limits Horizontal Gene Transfer in Staphylococci by Targeting DNA. Science.

[16] Zhang Q., Ye Y. (2017). Not all predicted CRISPR–Cas systems are equal: Isolated cas genes and classes of CRISPR like elements. BMC Bioinform..

[17] Palmer K.L., Gilmore M.S. (2010). Multidrug-Resistant Enterococci Lack CRISPR-cas. MBio.

[18] Price V.J., Huo W., Sharifi A., Palmer K.L. (2016). CRISPR-Cas and Restriction-Modification Act Additively against Conjugative Antibiotic Resistance Plasmid Transfer in *Enterococcus faecalis*. mSphere.

[19] Sycheva M.V., Kartashova O.L., Shchepitova N.E., Safronov A.A. (2016). Antibiotic Resistance of Enterococci Isolated from Healthy Humans and Patients with Various Pathologies. Antibiot. Khimioter..

[20] Marin Garrido A. (2014). Antimicrobial Resistance in Enterococci. J. Infect. Dis. Ther..

[21] Sivertsen A., Pedersen T., Larssen K.W., Bergh K., Rønning T.G., Radtke A., Hegstad K. (2016). A Silenced vanA Gene Cluster on a Transferable Plasmid Caused an Outbreak of Vancomycin-Variable Enterococci. Antimicrob. Agents Chemother..

[22] Wardal E., Żabicka D., Hryniewicz W., Sadowy E. (2022). VanA-*Enterococcus faecalis* in Poland: Hospital population clonal structure and vanA mobilome. Eur. J. Clin. Microbiol. Infect. Dis..

[23] Sun L., Chen Y., Hua X., Chen Y., Hong J., Wu X., Jiang Y., van Schaik W., Qu T., Yu Y. (2020). Tandem amplification of the vanM gene cluster drives vancomycin resistance in vancomycin-variable enterococci. J. Antimicrob. Chemother..

[24] Nishioka T., Ogawa W., Kuroda T., Katsu T., Tsuchiya T. (2009). Gene Cloning and Characterization of EfmA, a Multidrug Efflux Pump, from *Enterococcus faecium*. Biol. Pharm. Bull..

[25] Reynolds E.D., Cove J.H. (2005). Resistance to telithromycin is conferred by msr(A), msrC and msr(D) in Staphylococcus aureus. J. Antimicrob. Chemother..

[26] Belloso Daza M.V., Milani G., Cortimiglia C., Pietta E., Bassi D., Cocconcelli P.S. (2022). Genomic Insights of *Enterococcus faecium* UC7251, a Multi-Drug Resistant Strain From Ready-to-Eat Food, Highlight the Risk of Antimicrobial Resistance in the Food Chain. Front. Microbiol..

[27] Cordeiro-Moura J.R., Kraychete G.B., de Araújo Longo L.G., Corrêa L.L., da Silva N.M.V., Campana E.H., Oliveira C.J.B., Picão R.C. (2022). Description and comparative genomic analysis of a mcr-1-carrying *Escherichia coli* ST683/CC155 recovered from touristic coastal water in Northeastern Brazil. Infect. Genet. Evol..

[28] Tao S., Chen H., Li N., Fang Y., Xu Y., Liang W. (2022). Association of CRISPR-Cas System with the Antibiotic Resistance and Virulence Genes in Nosocomial Isolates of *Enterococcus*. Infect. Drug Resist..

[29] Pourcel C., Touchon M., Villeriot N., Vernadet J.-P., Couvin D., Toffano-Nioche C., Vergnaud G. (2019). CRISPRCasdb a successor of CRISPRdb containing CRISPR arrays and cas genes from complete genome sequences, and tools to download and query lists of repeats and spacers. Nucleic Acids Res..

[30] Alcock B.P., Huynh W., Chalil R., Smith K.W., Raphenya A.R., Wlodarski M.A., Edalatmand A., Petkau A., Syed S.A., Tsang K.K. (2023). CARD 2023: Expanded curation, support for machine learning, and resistome prediction at the Comprehensive Antibiotic Resistance Database. Nucleic Acids Res..

[31] Lyons C., Raustad N., Bustos M.A., Shiaris M. (2015). Incidence of Type II CRISPR1-Cas Systems in *Enterococcus* Is Species-Dependent. PLoS ONE.

[32] Tsegahun A. (2019). Biofilm Formation by *Enterococcus faecalis* and *Enterococcus faecium*: Review. Int. J. Res. Stud. Biosci..

[33] Tao S., Zhou D., Chen H., Li N., Zheng L., Fang Y., Xu Y., Jiang Q., Liang W. (2023). Analysis of genetic structure and function of clustered regularly interspaced short palindromic repeats loci in 110 *Enterococcus* strains. Front. Microbiol..

[34] Gholizadeh P., Aghazadeh M., Ghotaslou R., Rezaee M.A., Pirzadeh T., Cui L., Watanabe S., Feizi H., Kadkhoda H., Kafil H.S. (2021). Role of CRISPR-Cas system on antibiotic resistance patterns of *Enterococcus faecalis*. Ann. Clin. Microbiol. Antimicrob..

[35] Pursey E., Dimitriu T., Paganelli F.L., Westra E.R., van Houte S. (2022). CRISPR-Cas is associated with fewer antibiotic resistance genes in bacterial pathogens. Philos. Trans. R. Soc. B Biol. Sci..

[36] Price V.J., McBride S.W., Hullahalli K., Chatterjee A., Duerkop B.A., Palmer K.L. (2019). *Enterococcus faecalis* CRISPR-Cas Is a Robust Barrier to Conjugative Antibiotic Resistance Dissemination in the Murine Intestine. mSphere.

[37] dos Santos B.A., da Silva de Oliveira J., Parmanhani-da-Silva B.M., Ribeiro R.L., Teixeira L.M., Neves F.P.G. (2020). CRISPR elements and their association with antimicrobial resistance and virulence genes among vancomycin-resistant and vancomycin-susceptible enterococci recovered from human and food sources. Infect. Genet. Evol..

[38] Pinilla-Redondo R., Russel J., Mayo-Muñoz D., Shah S.A., Garrett R.A., Nesme J., Madsen J.S., Fineran P.C., Sørensen S.J. (2022). CRISPR-Cas systems are widespread accessory elements across bacterial and archaeal plasmids. Nucleic Acids Res..

[39] Pinilla-Redondo R., Mayo-Muñoz D., Russel J., Garrett R.A., Randau L., Sørensen S.J., Shah S.A. (2020). Type IV CRISPR–Cas systems are highly diverse and involved in competition between plasmids. Nucleic Acids Res..

[40] Murugesan A.C., Varughese H.S. (2022). Analysis of CRISPR–Cas system and antimicrobial resistance in *Staphylococcus coagulans* isolates. Lett. Appl. Microbiol..

[41] Garneau J.E., Dupuis M.-È., Villion M., Romero D.A., Barrangou R., Boyaval P., Fremaux C., Horvath P., Magadán A.H., Moineau S. (2010). The CRISPR/Cas bacterial immune system cleaves bacteriophage and plasmid DNA. Nature.

[42] Rodrigues M., McBride S.W., Hullahalli K., Palmer K.L., Duerkop B.A. (2019). Conjugative delivery of CRISPR-Cas9 for the selective depletion of antibiotic-resistant enterococci. Antimicrob. Agents Chemother..

[43] Varahan S., Hancock L.E. (2016). To Defend or Not To Defend: That’s the Question. mSphere.

[44] Huo W., Price V.J., Sharifi A., Zhang M.Q., Palmer K.L. (2023). *Enterococcus faecalis* Strains with Compromised CRISPR-Cas Defense Emerge under Antibiotic Selection for a CRISPR-Targeted Plasmid. Appl. Environ. Microbiol..

